# Simvastatin Induced Neurite Outgrowth Unveils Role of Cell Surface Cholesterol and Acetyl CoA Carboxylase in SH-SY5Y Cells

**DOI:** 10.1371/journal.pone.0074547

**Published:** 2013-09-11

**Authors:** Varshiesh Raina, Sarika Gupta, Saurabh Yadav, Avadhesha Surolia

**Affiliations:** 1 Molecular Sciences Laboratory, National Institute of Immunology, Aruna Asaf Ali Marg, New Delhi, Delhi, India; 2 Molecular Biophysics Unit, Indian Institute of Science, Bangaluru, Karnataka, India; Medical College of Wisconsin, United States of America

## Abstract

Statins are known to modulate cell surface cholesterol (CSC) and AMP-activated protein kinase (AMPK) in non-neural cells; however no study demonstrates whether CSC and AMPK may regulate simvastatin induced neuritogenesis (SIN). We found that simvastatin (SIM) maintains CSC as shown by Fillipin III staining, Flotillin-2 protein expression / localization and phosphorylation of various receptor tyrosine kinases (RTKs) in the plasma membrane. Modulation of CSC revealed that SIN is critically dependent on this CSC. Simultaneously, phospho array for mitogen activated protein kinases (MAPKs) revealed PI3K / Akt as intracellular pathway which modulates lipid pathway by inhibiting AMPK activation. Though, SIM led to a transient increase in AMPK phosphorylation followed by a sudden decline; the effect was independent of PI3K. Strikingly, AMPK phosphorylation was regulated by protein phosphatase 2A (PP2A) activity which was enhanced upon SIM treatment as evidenced by increase in threonine phosphorylation. Moreover, it was observed that addition of AMP analogue and PP2A inhibitor inhibited SIN. Bio-composition of neurites shows that lipids form a major part of neurites and AMPK is known to regulate lipid metabolism majorly through acetyl CoA carboxylase (ACC). AMPK activity is negative regulator of ACC activity and we found that phosphorylation of ACC started to decrease after 6 hrs which becomes more pronounced at 12 hrs. Addition of ACC inhibitor showed that SIN is dependent on ACC activity. Simultaneously, addition of Fatty acid synthase (FAS) inhibitor confirmed that endogenous lipid pathway is important for SIN. We further investigated SREBP-1 pathway activation which controls ACC and FAS at transcriptional level. However, SIM did not affect SREBP-1 processing and transcription of its target genes likes ACC1 and FAS. In conclusion, this study highlights a distinct role of CSC and ACC in SIN which might have implication in process of neuronal differentiation induced by other agents.

## Introduction

Statins are classic inhibitors of HMG CoA reductase, a rate limiting enzyme in mevalonate pathway involved in synthesis of cholesterol and isoprenoids [1]. Interestingly, statins promote neuritogenesis in neuroblastoma cells; however the precise mechanism behind neuritogenesis has remained enigmatic [2–4]. Commonly regarded as cholesterol lowering agents, studies show that statins tend to maintain cell surface cholesterol (CSC) in an asymmetric manner in non-neuronal cells [5]. The role of CSC in neuritogenesis is also evident from the fact that depletion of CSC in hippocampal and cortical neurons exerts differential effect on neurite outgrowth [6]. Furthermore, lipid composition of neurites revealed higher percentage of cholesterol than neuronal soma [7]. Additionally, there are studies which implicate importance of CSC in neuritogenesis in an indirect way [8–11].

Upcoming reports show that an AMP - activated protein kinase (AMPK) plays an important role in neuronal homeostasis [12,13]. Recently, a study showed that AMPK inhibits axon growth in hippocampal neurons. AMPK performs various biological functions within cells, including control of fatty acid metabolism by negatively regulating the activity of enzymes like Acetyl CoA carboxylase (ACC) and Fatty acid synthase (FAS) [12,14]. Fatty acids act as precursors for various phospholipids which are building blocks for neurites [15,16]. Remarkably, statins modulate AMPK activity in non-neuronal cells [17–19] and to our surprise no study has so far addressed the role of ACC in neuritogenesis. ACC is known to exist in two isoforms: ACC1 and ACC2 [20]. ACC1 is generally involved in fatty acid biosynthesis whereas ACC2 is involved in fatty acid catabolism. Transcriptionally, ACC is regulated by a Sterol Response Element Binding Protein-1 (SREBP-1), which is also regarded as a target of AMPK [20–22]. Like other SREBPs, SREBP-1 is bound to endoplasmic reticulum as inactive precursors and once processed the active form enters the nucleus for transcription of target genes. Interestingly, statins have been shown to modulate SREBP processing in non-neuronal cells [23–25]. In addition, studies show that application of exogenous fatty acids strongly stimulates neuritogenesis [26,27]. Surprisingly, till date no study has investigated the role of endogenous lipid modulators during the process of neuritogenesis.

In this study, we were interested to find out whether membrane cholesterol and AMPK / ACC pathway play any role in simvastatin induced neuritogenesis (SIN). We choose simvastatin (SIM) because of its well known role as a therapeutic agent in various neurological diseases and inducer of neuritogenesis. SH-SY5Y cells were used as target cells because of their ability to develop well differentiated neurites. We show for the first time that SIM modulates CSC and activity of ACC for inducing neuritogenesis in SH-SY5Y cells.

## Materials and Methods

### Chemicals and antibodies

Inhibitors PD98059, LY294002, SP600125, Rapamycin, Pifithrin α, SB203580, Protein Kinase A inhibitor fragment 14-22, Fostriecin, Cyclodextrins like MβD, α-Cyclodextrin and γ-Cyclodextrin; Cholesterol; Mevalonic acid; U 18666a; GGTI-298; AICAR; TOFA; Cerulenin; 25-hydroxycholesterol; Filipin III; α-Lysophosphatidylcholine; and Simvastatin were obtained from Sigma (St. Louis, MO, USA). The antibody against GAP43, Nestin, Neurofilament-L, Flotillin-2, SREBP-1, Actin and HRP-conjugated secondary antibody were obtained from Santa Cruz Biotechnology (2145 Delaware Avenue, CA, USA) whereas antibody against PP2C, p-Tyrosine hydroxylase, AMPKα, pAMPKα (Thr172), ACC, pACCser79 and β3-tubulin were obtained from Cell Signaling Technology (Beverly, MA, USA).

### Filipin III staining for cholesterol detection

Filipin labeling of membrane cholesterol was performed as previously described [28]. Briefly, SH-SY5Y cells were seeded and cultured in 30mm petri plates on poly l-lysine coated coverslips. Cells were rinsed 3 times with Dulbecco’s PBS (pH 7.4, GIBCO) and fixed for 30 minutes in 4% PFA in PBS containing 0.12 mM sucrose and labeled with 300 µg/ml filipin (Sigma) in PBS for 90 min. After washing with PBS, the cells were fixed for a second time in PFA for 20 min and mounted on a microscope slide using 90% (v/v) glycerol in PBS. Filipin fluorescence was analyzed by taking images at 60 X magnification and 5 s exposures using MetaMorph Image Analysis software (Universal Imaging) and a Photometrics, Cool Snap HQ2 camera coupled to a fluorescent microscope (Nikon Diaphot).

### Immunoblotting and Immunoprecipitation

Whole-cell lysates were prepared from SH-SY5Y cells that had been incubated with or without SIM by harvesting them in NP-40 lysis buffer containing: 1% NP40, 50 mM Tris-Cl pH 8, 150 mM NaCl, 2 mM EDTA, 1 mM DTT, 1 mM NaF, 100 µM Na _2_VO_4_ and protease inhibitor cocktail (Roche, Basel, Switzerland). Protein estimation was done with Bradford reagent (BioRad, Alfred nobel drive Hercules, CA, USA) using Magellan V 6.6 software in TECAN Infinite M200 machine. 50µg of whole cell lysate was mixed with Laemmli loading buffer and boiled for 10 min, after which they were loaded and run on SDS-polyacrylamide gels as previously described [29]. The proteins were transferred to nitrocellulose membranes (Protran, Whatman, Kent, UK) using Biorad (Hercules, CA, USA) electrophoresis systems onto Immobilon membranes (Millipore Bioscience Research Reagents) and immunoblotted (Towbin et al., 1979; Ferreira et al., 1997) with primary and secondary antibodies. The proteins were detected with ECL reagent (Pierce) in LAS-4000 from Fujifilm. Density of proteins bands was analyzed by Image reader software from Fujifim.

For immunoprecipitation, cells were lysed with RIPA buffer (Sigma) and the supernatant was precleared with appropriate volume of protein A/G PLUS-Agarose beads (Santa Cruz Biotechnology, Inc). Approximately, 500 µg of the total protein was incubated with 2.0 µg of primary antibody for 1 hr at 4^0^C. Now 20 µl of protein A/G PLUS-Agarose beads were added and samples were allowed to incubate overnight at 4^0^C on a rotating device. Immunoprecipitate was collected by centrifugation at 2,500 r.p.m for 5 minutes at 4^0^C. The pellet was washed; suspended in 1x electrophoresis sample buffer; boiled for 3 minutes and subjected to SDS-PAGE. After transfer to nitrocellulose membrane the protein of interest was detected by appropriate primary and secondary antibodies.

### Immunofluorescence microscopy

Cells cultured on glass cover slips were fixed in 3.7% paraformaldehyde (10 min at room temperature), blocked with 0.5%. BSA and 0.2% gelatin in PBS, and then probed with Flotillin-2 primary antibody and FITC-labeled secondary antibody (Sigma). DNA was counterstained with DAPI (Sigma Aldrich). Confocal images were obtained in Zeiss LSM510 META laser-scanning confocal microscope. A Pan-Apochromat 63X/1.4 oil-immersions DIC M27 objective (Zeiss) was used for image acquisition with a Leica TCS SP2 AOBS confocal laser microscope by sequential scanning.

### Phospho Receptor Tyrosine Kinase (p-RTK) and phospho MAP Kinase array (p-MAPK)

Protein array kits for p-RTK and p-MAPK were obtained from Cell Signaling and the proteins were assessed according to the manufacturer instructions.

### Thin Layer Chromatography of cholesterol

In order to estimate the cholesterol content control and simvastatin treated SH-SY5Y cells were frozen at -20°C in 1 mL PBS and then the samples were thawed and extracted with methanol-chloroform mixture (2:1, v/v). The mixture was shaken for 2 hrs at room temperature (RT). The supernatants were transferred to a Teflon-stoppered tube after centrifugation. The residual pellet was re-extracted with methanol-chloroform-water (2:1:0.8, v/v) and centrifuged. The supernatants from the two extractions were combined, diluted with chloroform-water (1:1), centrifuged and the organic layer (bottom) removed before drying under N_2_. The residue was reconstituted with 50 µL of chloroform. Samples were spotted on a Whatman LK6 TLC silica plate with pre-adsorbent strip (VWR, Buffalo Grove, IL, USA). TLC was performed using a one-tank solvent system [30]. The plates were run in benzene-ethyl acetate (3:2). The plates were dried at room temperature and developed by spraying a mixture of cupric acetate (3% w/v) and phosphoric acid (8% w/v), then charred in an oven at 120°C for 30 min [31]. The plates were then scanned and the results analyzed using National Institutes of Health Image v.1.44 software (NIH, Bethesda, MD, USA).

### Reverse transcriptase Polymerase chain reaction

Total RNA was extracted from SH-SY5Y cells with Trizol Reagent as per the manufacturer’s instructions. Concentrations of total RNA were measured by spectrophotometry (Nanodrop ND-100 spectrophotometer from Biolabs). Total RNA was reverse transcribed in a reaction mix containing following final concentration of reagents: 1X M-MuLV Reverse Transcriptase Reaction Buffer, 5.5 mM MgCl_2_, 2 mM dNTP mixture (500 µM of each dNTP), 2.5 µM random hexamers, 0.4 units/µl RNase inhibitor, 200 units M-MuLV Reverse Transcriptase, ^~^1.0 µg RNA, and RNase-free water to a total volume of 25.0 µl. To conduct reverse transcriptase thermal cycling, the samples were run in S1000 thermal cycler (BioRad) under the following conditions: incubation for 10 minutes at 25°C, reverse transcription for 45 minutes at 42°C, and inactivation of reverse transcriptase for 5 minutes at 80°C. All complementary DNA (cDNA) samples were stored at −20°C. For polymerase chain reaction around 1-2 µl of cDNA was used as template in a reaction mixture containing 10 mM Tris-HCl (pH 8.3), 50 mM KCl, 1.5 mM MgCl_2_, 200 µM each of dNTP, 20 pmol of each primer (Table 1) and 2U of Taq DNA polymerase. Reactions were initiated by the incubation at 94°C for 5 min, and PCR (denaturation at 94°C for 30 sec, annealing at 60°C for 40 sec, and extension at 72°C for 45 sec, respectively) was performed for 35 cycles with a final extension at 72°C for 10 min. Electrophoresis was run for an aliquot of PCR amplification products on a 2% (w/v) agarose gel, followed by the detection of DNA with ethidium bromide.

**Table 1:** Forward and reverse Primer sequences of the following genes: FAS, ACC1, ACC2 and β-actin along with the expected product size in base pairs.

**Genes**	**Upstream Primer (5’ -3**’)	**Downstream primers (5’ -3**’)	**Amplicon**
FAS	GTGAGGCTGAGGCTGAGAC	GGCACGCAGCTTGTAGTAGA	211
ACC1	TTAACAGCTGTGGAGTCTGGCTGT	AACACTCGATGGAGTTTCTCGCCT	187
ACC2	GGTGCTTATTGCCAACAACGGGAT	TCTTGATGTACTCTGCGTTGGCCT	151
β-Actin	CTGGGACGACATGGAGAAAA	AAGGAAGGCTGGAAGAGTGC	564

### Statistics analysis

The values are expressed as the mean ± standard error of the mean (S.E.M). Comparisons between the different treatment groups were analyzed via one-way ANOVA and the least significant difference (LSD), and differences were considered significant at p < 0.05^*^ and highly significant at p < 0.001.

## Results

### SIM promotes axon like neuritogenesis in SH-SY5Y cells

**Figure 1:**
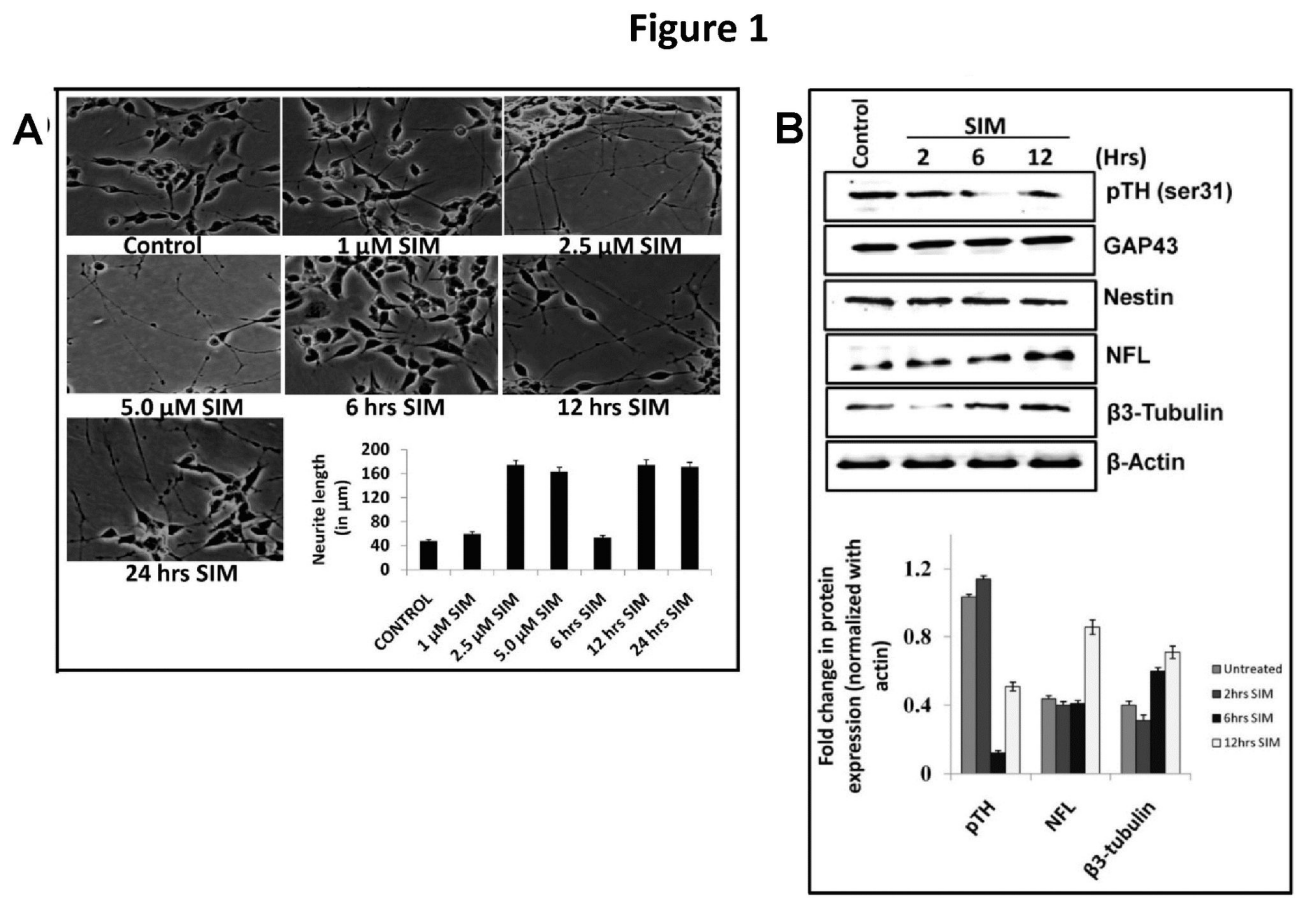
SIM promotes axon like neuritogenesis in SH-SY5Y cells. A) Morphology of SH-SY5Y under light microscope (Nikon Eclipse, 80i; 10x magnification) showing neuritogenic effect of SIM as function of concentration and time. SH-SY5Y cells were treated with SIM at concentration of 1.0 µM, 2.5 µM and 5.0 µM for a period of 24 hrs. A significant (*p* < 0.001) increase in neuritogenesis occurred at concentration of 2.5 µM SIM. Error bar graph represents the difference in neurite length (in μm) between control and SIM treated cells (mean ± SEM; N = 50 per condition from 3 separate cultures). Treatment of SH-SY5Y cells with 2.5 µM SIM for different time periods i.e. 6 hrs, 12 hrs and 24 hrs showed that 12 hrs time was the minimal time point for inducing significant (*p* < 0.001) increase in neurite length. Error bar graph represents the difference in neurite length (in μm) between control and SIM treated cells (mean ± SEM; N = 50 per condition from 3 separate cultures). Scale Bar = 100µm. **B**) Immunoblot showing SIM induced change in protein expression of neuronal differential markers like neurofilament L (NFL), β3-tubulin, GAP43 and Nestin. Note that SIM led to a significant (*p* < 0.05^*^) increase in protein expression of NFL and β3-tubulin whereas GAP43 and Nestin displayed no change. phospho-Tyrosine Hydroxylase serine 31 (pTH-Ser31), a dopaminergic neuronal functional marker showed a significant (*p* < 0.05^*^) decrease in protein levels at 6 hrs post SIM treatment. Error bar graph represents the fold change in protein expression of pTH, NFL and β3-tubulin normalized to loading control i.e. actin (mean ± SEM; n = 4).

Though action of statins on neuritogenesis is known [2–4]; however the detailed mechanism of simvastatin induced neuritogenesis has remained unexplored. Treatment of SH-SY5Y cells (a dopaminergic cell line) with different concentrations of Simvastatin (SIM) for 24 hrs showed that 2.5 µM of SIM was the minimum concentration to promote significant increase in neuritogenesis and time kinetics revealed that significant increase in neuritogenesis occurred at 12 hrs (Figure **1A**). Morphologically, bead like structures on neurites indicated axon like outgrowth. To confirm axonal outgrowth two important markers i.e. Neurofilament L (which indicates radial axon growth and determine axon caliber) and β3-tubulin (which indicates axon outgrowth) were analyzed. Remarkably, SIM enhanced the protein expression of β3-tubulin (nearly 2 fold vs. control) and NFL (greater than 2 fold vs. control). We also assessed the expression of nestin and GAP43 proteins which are commonly used as markers of neuronal differentiation. However, after SIM treatment neither nestin nor GAP43 showed any change in protein levels (Figure **1C**) which clearly indicates that SIM only promotes neuritogenesis. Tyrosine hydroxylase (TH), which is considered as a functional marker of dopaminergic neurons was also evaluated. An increase in Ser-31 phosphorylation of TH determines the activity of this enzyme. Though SIM led to a drastic decrease in TH (Ser-31) levels at 6 hrs; however at 12 hrs TH (Ser-31) levels showed some reversal in dephosphorylation (Figure **1B**).

### Retention of cell surface cholesterol maintains neuritogenic effect of SIM

**Figure 2:**
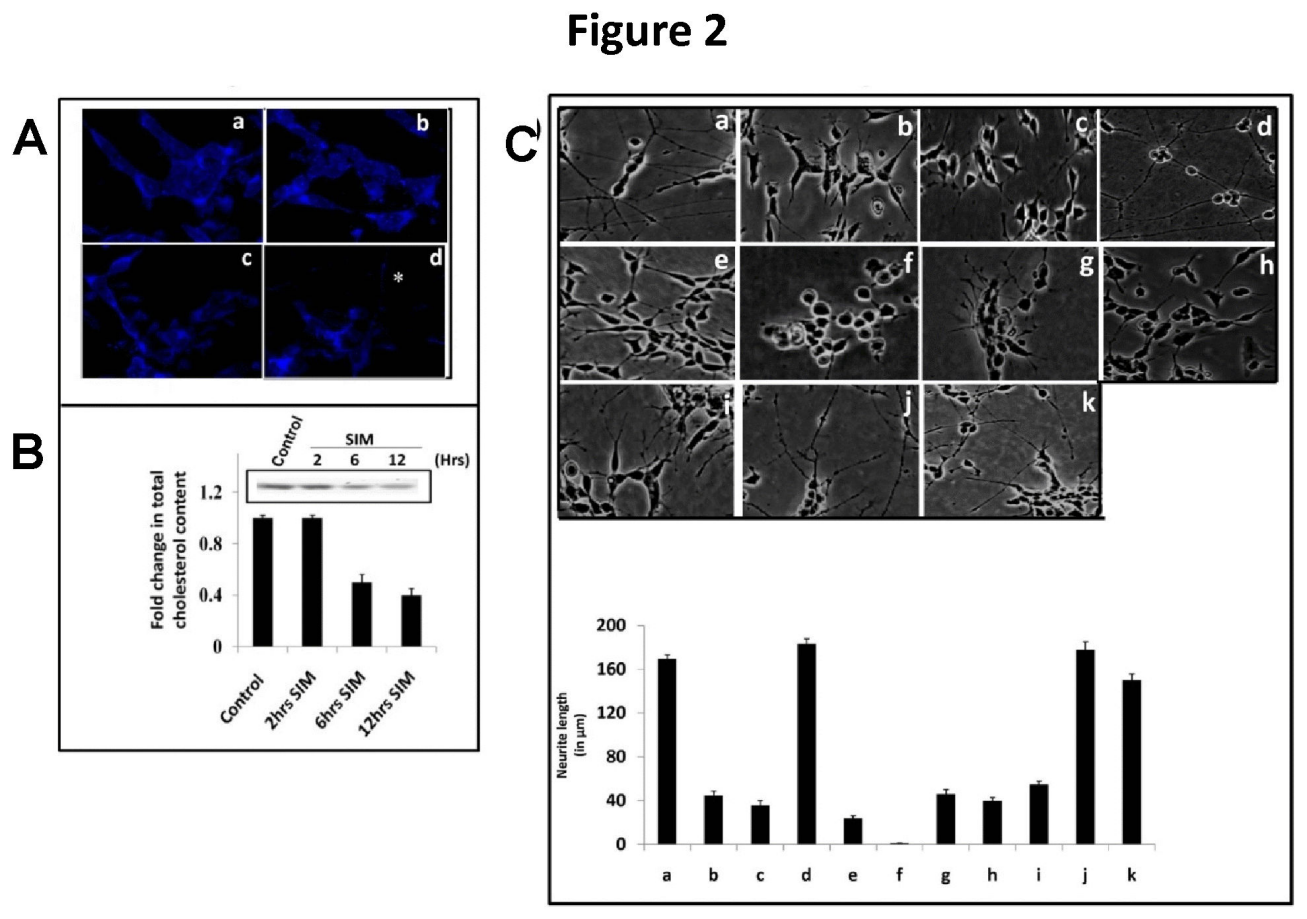
Retention of CSC in plasma membrane determines SIM induced neuritogenesis in SH-SY5Y cells. **A**) Confocal image of SH-SY5Y cells at 60 X magnification showing fluorescence of cholesterol binding probe i.e. Filipin III in control (a); and SIM treated cells for 2 hrs (b), 6 hrs (c) and 12 hrs (d). The fluorescence (shown as blue fluorescence) was observed not only in plasma membrane but also in neurites. The neurite has been represented by *. N = 25-30 per condition from 3 separate cultures (n = 3). Scale Bar = 10 µm. **B**) Thin layer chromatography showing total cellular cholesterol content at 2 hrs, 6 hrs and 12 hrs after SIM treatment. A significant (*p* < 0.001) decrease in cholesterol content occurred after 6 hrs. Difference in cholesterol content is represented in error bar graph calculated as percent change (mean ± SEM; n = 4). **C**) Morphology of SH-SY5Y cells under light microscope (10 X magnification) showing the neuritogenic effect of SIM in presence of agents which perturb cholesterol function. Cells were treated with SIM alone (a); or along with compounds like 200 µM MβD, cholesterol scavenger (b); 10 µM U18666a, intracellular cholesterol transport inhibitor (c); 15 µg/ml cholesterol (chol.) complexed with 200 µM MβD (d); 0.5 mM mevalonate (Meva.), a precursor for cholesterol biosynthesis (e); 10 µM GGTI-298, an inhibitor of Geranylgeranyltransferase (f); 5 µg/ml Filipin III, a cholesterol binding probe (g); and 10 µg/ml α-lysophosphatidylcholine (α-LPC), a cholesterol intercalater (h) after 1 hr post SIM treatment. Neuritogenesis induced by SIM was significantly (*p* < 0.05^*^) reduced by MβD, U 18666a, mevalonate, GGTI-298, Filipin III and α-LPC whereas exogenous cholesterol supplementation did not change significantly the neuritogenic effect of SIM. Concomitantly, SIM treated SH-SY5Y cells were incubated along with sphingomyelin, SPM (i); and with inhibitors which modify endogenous sphingolipid biosynthesis like 10 µM GW4869, an inhibitor of neutral sphingomyelinase (j) and Fumonisin B1, an inhibitor for ceramide synthase (k). Application of exogenous SPM led to a significant (*p* < 0.001) decrease in SIM induced neuritogenesis whereas modulation of endogenous sphingolipid pool produced no significant effect on neuritogenic effect of SIM. The change in neurite length (in μm) is represented by error bar graph (mean ± SEM; N = 40-65 cells per condition from 3 separate cultures). Scale Bar = 100 µm.

Earlier reports showed that like other statins simvastatin retains cholesterol on plasma membrane in non-neural cells [5]. However, it is unclear whether simvastatin retains cell surface cholesterol (CSC) in neuronal cells like SHSY5Y. We therefore examined the effect of SIM on cholesterol in SH-SY5Y by two methods: Filipin III labeling which detects cholesterol distribution in cell and thin layer chromatography for determining cholesterol concentration. Filipin III labeling showed that SIM did not alter much cholesterol distribution on plasma membrane till 6 hrs. At 12 hrs cholesterol was partly distributed in plasma membrane and partly in neurites (Figure **2A**). Simultaneously, thin layer chromatography revealed that SIM decreased cholesterol concentration by almost 2 fold from 6 hrs onwards (Figure **2B**).

We next wanted to investigate whether CSC plays any role in maintaining neuritogenesis. To investigate the role of CSC, some agents were employed which are known to perturb function or integrity of cholesterol in plasma membrane like methyl-beta cyclodextrin (MβD; a well known surface cholesterol scavenger), Filipin III (cholesterol sequester), α-lysophosphatidylcholine (α-LPC; a natural cholesterol intercalater) and U-18666a (an intracellular cholesterol transport inhibitor). Addition of different concentrations of MβD (**not shown here**) revealed 200 μΜ MβD as a minimal effective concentration to inhibit SIN (Figure **2C**). Also, MTT assay showed that at this concentration (i.e. 200 µM) MβD had no effect on cell viability in the presence of SIM (**not shown here**). Other cyclodextrins like α-Cyclodextrin (α-CD) and γ-Cyclodextrin (γ-CD) known to poorly extract surface cholesterol showed no significant effect on SIN (**not shown here**). Likewise, application of U-18666a also inhibited SIN (Figure **2C**). Cholesterol plays an important role in plasma membrane to regulate its fluidity; we thus perturbed the CSC by applying two agents like Filipin III and α-LPC which block interaction of cholesterol in its physiological form. As shown in Figure **2C**, both Filipin III and α-LPC also reduced the SIN. Importantly, supplementing the cells with cholesterol loaded MβD did not show any effect on SIN (Figure **2C**). As statins block mevalonate pathway, we thus added mevalonate to observe whether SIN gets reversed. Expectedly, exogenous addition of mevalonate reversed the neuritogenic effect of SIM (Figure **2C**). Thus the data clearly indicates that retention of CSC but not the continuous synthesis of cholesterol is required for SIN. Mevalonate also acts as a precursor for isoprenoids in mevalonate pathway. While addition of Geranylgeranyltransferase (GGT) inhibitor i.e. GGTI-298 alone did not affect neuritogenesis (**not shown here**); however GGTI-298 completely suppressed SIN and cells became rounded (Figure **2C**).

Within plasma membrane CSC exists in association with sphingolipid in discrete domains as lipid rafts; however addition of major neuronal sphingolipid i.e. sphingomyelin reduced the SIN (Figure **2C**). Importantly, addition of inhibitors for neutral sphingomyelinase (GW4869) and ceramide synthase (Fumonisin B1) revealed no significant change in neurite outgrowth compared to SIM (Figure **2C**). Taken together, these data demonstrate that CSC plays an independent role in determining SIM induced neurite outgrowth.

### SIM retained CSC enhances the phosphorylation of various p-receptor tyrosine kinases (p-RTKs) and signaling through PI3K/Akt pathway

**Figure 3:**
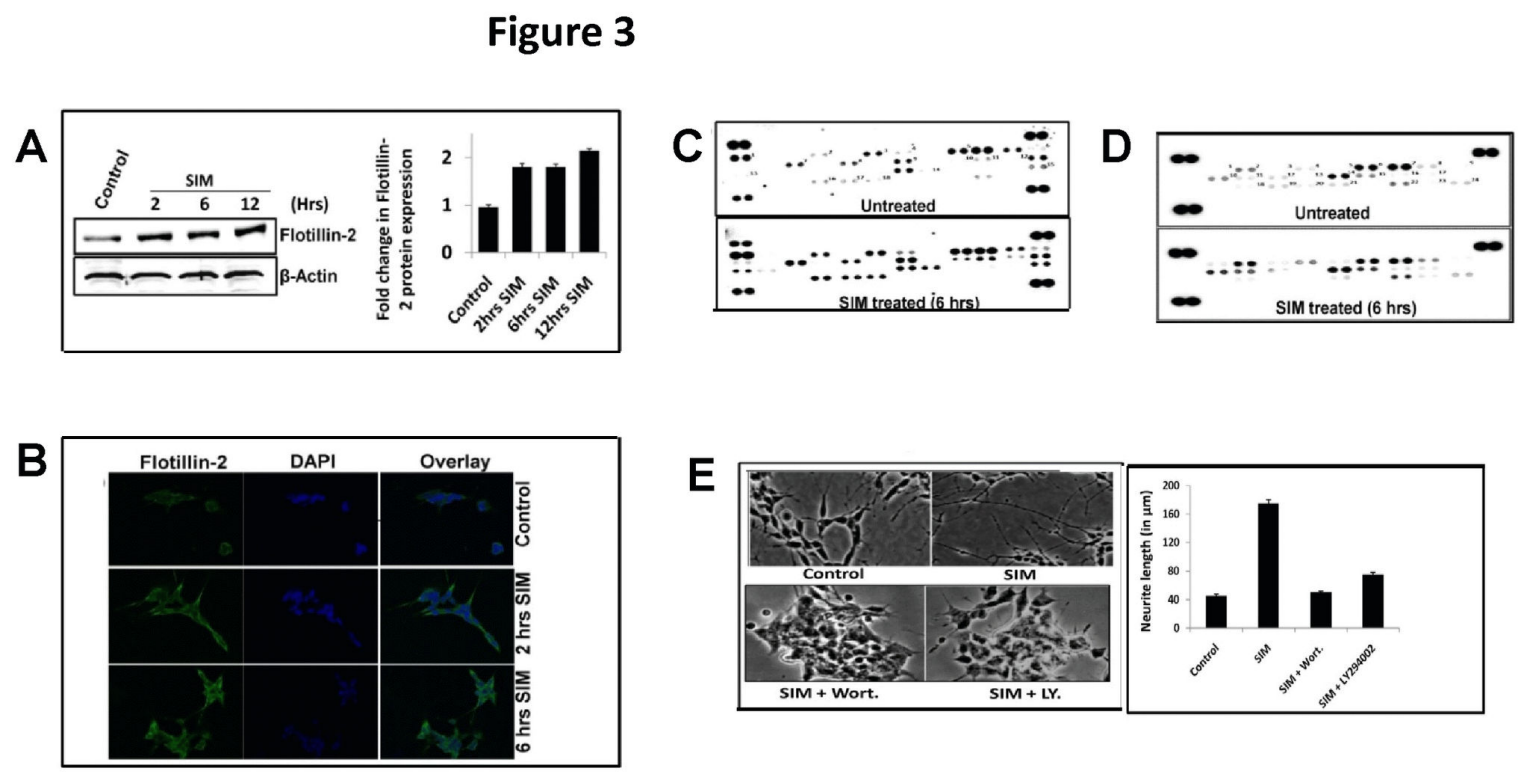
SIM retained CSC modifies various RTK’s and MAPK’s during neuritogenesis. A) Immunoblot showing significant (*p* < 0.05^*^) increase in protein levels of Flotilin-2 (a lipid raft marker) after SIM treatment from 2 hrs onwards. Error bar graph represents the fold change in protein levels with respect to untreated sample (mean ± SEM; n = 3). **B**) Confocal image at 60 X magnification showing marked increase in surface distribution of Flotilin-2 protein after SIM treatment till 6 hrs. For detection of Flotilin-2 protein, FITC-labeled secondary antibody was used which is shown as green fluorescence. Scale Bar = 10 µm. **C**) p-RTK protein array blot showing detection of various kinases represented as dots. Each kinase has been denoted by number as shown in blot and corresponding fold change in phosphorylation (ratio of SIM treated and untreated cells) at 6 hrs has been shown on right hand side of blot. The result is average of two experiments. Increased phosphorylation of various RTK’s like EGFR, ErbB4, FGFR2α, Insulin R, Dtk, MSPR, PDGF Rβ, Flt-3, c-Ret, ROR1, ROR2, Tie-2, MUSK, Eph B2, Eph A4, Eph B4 and Eph B6 was observed after SIM treatment **D)** p-MAPK protein array blot showing detection of various kinases represented as dots. Each kinase has been denoted by number as shown in blot. **E**) Morphology of SH-SY5 cells at 10 X magnification under light microscope showing significant (*p* < 0.001) reduction in neuritogenesis after addition of PI3K inhibitors i.e. wortmannin (Wort.) and LY294002 (LY.) at 12 hrs post SIM treatment. Each inhibitor was used at a concentration of 10 µM. The change in neurite length (in μm) is represented by error bar graph (mean ± SEM; N = 50 cells per condition from 3 separate cultures). Scale Bar = 100 µm.

One of the major functions of CSC is to maintain integrity of lipid rafts which transduce signals to regulate various biological processes like neuronal differentiation. The presence of cholesterol rich lipid rafts in cells is generally demonstrated by the presence of Flotillin-2 protein and phosphorylation of Receptor Tyrosine Kinases (RTKs). Treatment of SHSY5Y cells with SIM led to a marked increase not only in of Flotillin-2 protein expression (Figure **3A**) but also surface localization which was quite evident after 2 hrs (Figure **3B**). To examine the functionality of this CSC, phospho-Receptor Tyrosine Kinase (p-RTK) protein array was performed at 2 hrs and 6 hrs. No significant change in p-RTK phosphorylation was observed between samples at 2 hrs (**not shown here**). However, at 6 hrs SIM enhanced phosphorylation of various RTKs like EGFR (2.35 fold), ErbB4 (1.82 fold), FGF R2α (1.67 fold), FGF R3 (1.23 fold), Insulin R (2.2 fold), Dtk (1.3 fold), MSPR (2.39 fold), PDGF Rβ (1.8 fold), Flt-3 (2.2 fold), c-Ret (1.5), ROR1 (3.03 fold), ROR2 (1.28), Tie-2 (2.89 fold), MUSK (3.0 fold), Eph A4 (2.65), Eph B2 (3.09 fold), Eph B4 (2.53 fold) and Eph B6 (2.59 fold) (Figure **3C** and Table **2**) which clearly shows that SIM maintains the integrity of cholesterol rich rafts. To find out the intracellular signaling pathway which takes part in RTK signaling, we thus further performed a phospho-MAP Kinase (p-MAPK) protein array at 6 hrs. As shown in (Figure **3D** and Table **3**) SIM induced a significant increase in the phosphorylation of Akt2 kinase (2.0 fold) and HSP27 (1.5) whereas other kinases or MAPK targets like Akt1 (1.3 fold), Akt3 (1.2 fold), Akt pan (1.25 fold), CREB (1.22 fold), GSK-3α/β (1.29 fold), GSK-3β (1.0 fold), HSP27 (1.5 fold), JNK1 (1.3 fold), JNK2 (1.0 fold), JNK3 (1.0 fold), JNK pan (1.27 fold), MKK3 (1.32 fold), MKK6 (1.32 fold), p38β (1.13 fold), P53 (0.95 fold), RSK1 (1.15 fold), RSK2 (1.06 fold) and TOR (1.03 fold) showed minor change in phosphorylation. In addition phosphorylation of ERK1 (0.87), ERK2 (0.8) p38δ (0.66 fold) and P38γ (0.74) was markedly reduced after SIM treatment (Figure **3D**). Taken together our MAPK data indicates that activation of RTK’s by SIM involves PI3K/Akt pathway during neuritogenesis. Indeed, addition of PI3K inhibitor’s like LY294002 (10 µM) and Wortmannin (10 µM) completely abolished the SIN (Figure **3E**).

**Table 2:** Corresponding fold change in phosphorylation of various receptor tyrosine kinases (RTK’s) in presence and absence of SIM in SH-SY5Y at 6 hrs.

**S. No**	**p-RTK’s**	**Fold Change (SIM treated / Untreated**)
1	EGF-R	2.35
2	Erb B4	1.82
3	FGF R2α	1.67
4	FGF R3	1.23
5	Insulin R	2.2
6	Dtk	1.3
7	MSP R	2.39
8	PDGF Rβ	1.8
9	Flt3	2.2
10	c-Ret	1.5
11	ROR1	3.03
12	ROR2	1.28
13	Tie2	2.89
14	MuSK	3.0
15	Eph A4	2.65
16	Eph B2	3.09
17	Eph B4	2.53
18	Eph B6	2.59

The result is average of two experiments. Increased phosphorylation of various RTK’s like EGFR, ErbB4, FGFR2α, Insulin R, Dtk, MSPR, PDGF Rβ, Flt-3, c-Ret, ROR1, ROR2, Tie-2, MUSK, Eph B2, Eph A4, Eph B4 and Eph B6 was observed after SIM treatment. The result is mean of two experiments with standard deviation less than 5%.

**Table 3:** Corresponding fold change in phosphorylation of various Mitogen Activated Protein Kinases (MAPK’s) in presence and absence of SIM in SH-SY5Y cells at 6 hrs.

**S. No**	**p-MAPK’s**	**Fold Change (SIM treated / Untreated**)
1	Akt1	1.3
2	Akt2	2.0
3	Akt3	1.2
4	Akt pan	1.25
5	CREB	1.22
6	ERK1	0.87
7	ERK2	0.8
8	GSK-3α/β	1.29
9	GSK-3 β	1.0
10	HSP27	1.5
11	JNK1	1.3
12	JNK2	1.0
13	JNK3	1.0
14	JNK pan	1.27
15	MKK3	1.32
16	MKK6	1.1
17	p38β	1.13
18	p38δ	0.66
19	p38γ	0.74
20	p53	0.95
21	RSK1	1.15
22	RSK2	1.06
23	TOR	1.03

A 2 fold increase in Akt2 phosphorylation was observed whereas other MAPKs showed a minor change. The result is mean of two experiments with standard deviation less than 5%.

### SIM induced neuritogenesis involves AMPK dephosphorylation through activation of PP2A phosphatase and not PI3K

**Figure 4:**
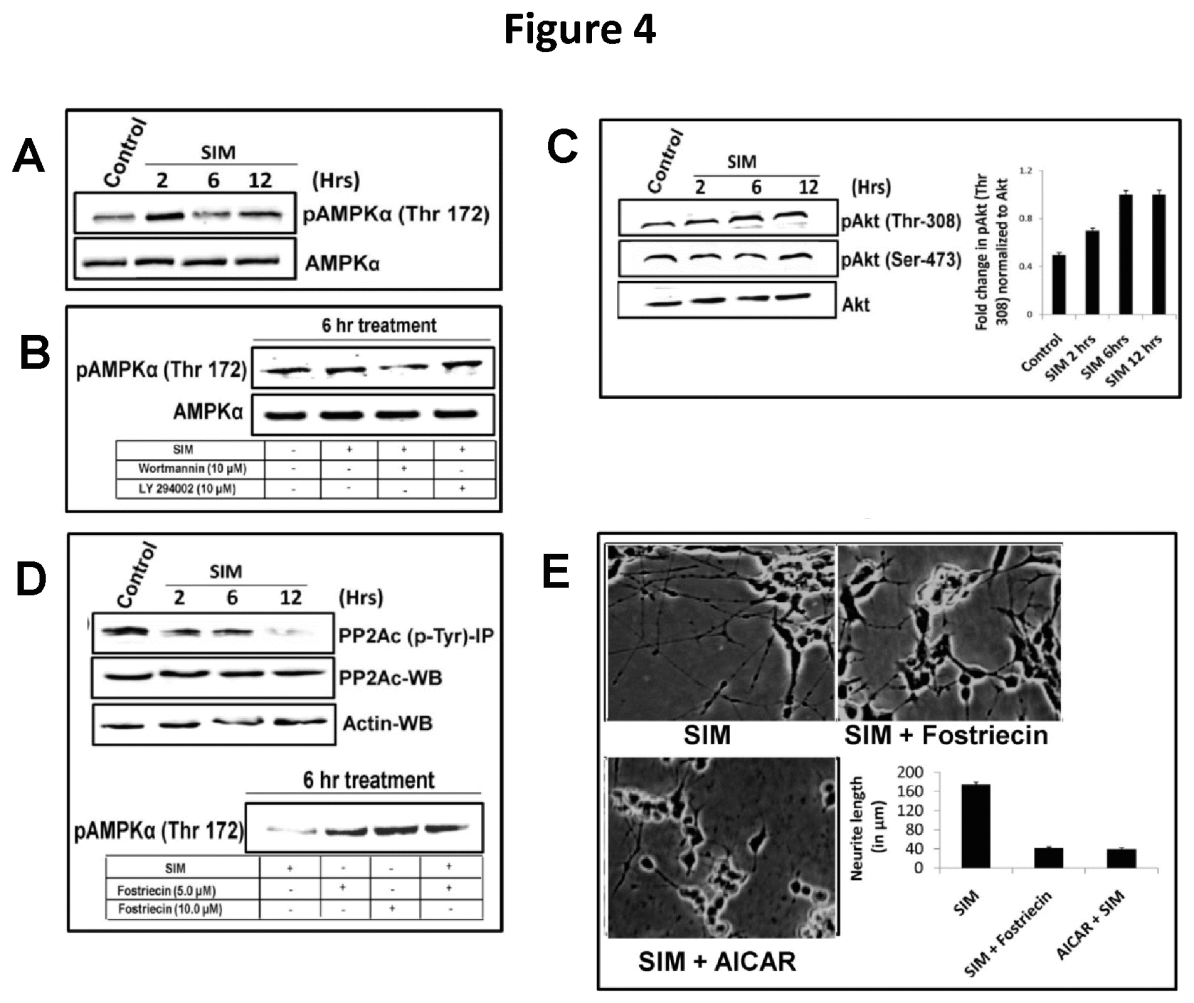
Dephosphorylation of AMPK by PP2A phosphatase promotes neuritogenic effect of SIM in SH-SY5Y cells. Immunoblot in Figure **3 (A, B, C and D)** showing modulation of protein expression of AMPK, p-AMPK (Thr-172), Akt, pAkt (ser-473), pAkt (Thr-308), PP2Ac and PP2Ac (p-Tyr) after SIM treatment for 12 hrs. **A**) protein expression of AMPK and p-AMPK (Thr-172) at 2 hrs, 6 hrs and 12 hrs after SIM treatment. At 2 hrs a sudden increase in phosphorylation of AMPK was noted which was accompanied by rapid reversal to control. **B**) AMPK and p-AMPK (Thr-172) levels at 6 hrs in presence of SIM and PI3K inhibitors like wortmannin and LY294002, 10 µM each. No change in pAMPK (Thr-172) was observed among the samples. **C**) Akt, pAkt (ser-473) and pAkt (Thr-308) levels at 2 hrs, 6 hrs and 12 hrs after SIM addition. A significant (*p* < 0.05^*^) 2.0 fold increase in pAkt (Thr-308) was observed whereas pAkt (Ser-473) displayed no change. **D**) Time dependent decrease in tyrosine phosphorylation of PP2Ac after SIM treatment till 12 hrs. Total PP2Ac protein levels remained same during SIM treatment. Simultaneously, inhibition of PP2A activity by Fostriecin showed increase in pAMPK (Thr-172) levels in presence or absence of SIM at 6 hrs **E**) Morphology of SH-SY5Y cells at 10 X magnification under light microscope showing significant (*p* < 0.001) decline in neuritogenic effect of SIM in presence of Fostriecin and an AMP analogue i.e. AICAR. Error bar graph shows the difference in neurite length between SIM, SIM + Fostriecin and SIM + AICAR treated cells for 12 hrs (mean ± SEM; N = 50 cells per condition from 3 separate cultures). Scale Bar = 100 µm.

PI3K/Akt pathway has critical regulatory role in controlling diverse cellular processes, including cellular homeostasis through inhibition of AMP-activated protein kinase (AMPK), a serine / threonine kinase [13,32]. Treatment of SH-SY5Y cells with SIM revealed a marked increase in the phosphorylation of AMPK (Thr-172) at 2 hrs followed by a sudden decline to basal level (Figure **4A**). On the other hand total AMPK protein levels showed no change (Figure **4A**). To delineate whether PI3K/Akt pathway is involved in regulation of AMPK phosphorylation, cells were treated with SIM along with two kinds of PI3K inhibitors: wortmannin (irreversible inhibitor) and LY294002 (reversible inhibitor). While LY294004 produced no effect on AMPK phosphorylation; wortmannin led to some decline in AMPK phosphorylation as observed at 6 hrs (Figure **4B**). Akt is a serine/threonine kinase which gets fully activated when both the residues are phosphorylated [33]. Our p-MAPK array data doesn’t delineate between these two phosphorylation sites. We thus performed an immunoblot for p-Akt (ser-473) and p-Akt (Thr-308) and observed that SIM did not change the p-Akt (Ser-473) levels whereas p-Akt (Thr-308) levels showed a significant 2.0 fold increase (Figure **4C**). This clearly indicates that SIM does not activate PI3K/Akt pathway for dephosphorylation of AMPK.

As shown in Figure **4B**, addition of wortmannin potentiated the dephosphorylation of AMPK along with SIM. This led us to hypothesize that silencing of Akt activity might be activating some phosphatase. We suspected PP2A phosphatase in this context as it is highly abundant in neuronal cells and it regulates phosphorylation of MAPKs (like Akt and ERK) as well as AMPK [34–36]. Tyrosine phosphorylation of PP2A in its catalytic subunit i.e. PP2Ac has been implicated to inhibit the activity of PP2A [37,38]. SIM led to a gradual decline in the tyrosine phosphorylation of PP2Ac whereas total PP2Ac protein levels remained unaltered (Figure **4D**). Addition of a PP2A inhibitor i.e. Fostriecin (10 µM) strikingly enhanced the AMPK phosphorylation along with SIM as observed at 6 hrs (Figure **4D**). Moreover, addition of Fostriecin abolished the SIN (Figure **4E**). Thus our result indicates that activation of AMPK has negative impact on neuritogenesis. Indeed, addition of a potent AMPK activator i.e. AICAR declined SIN and cells became rounded (Figure **4E**).

### Activation of an AMPK substrate i.e. acetyl CoA carboxylase (ACC) is essential for neuritogenic effect of SIM

**Figure 5:**
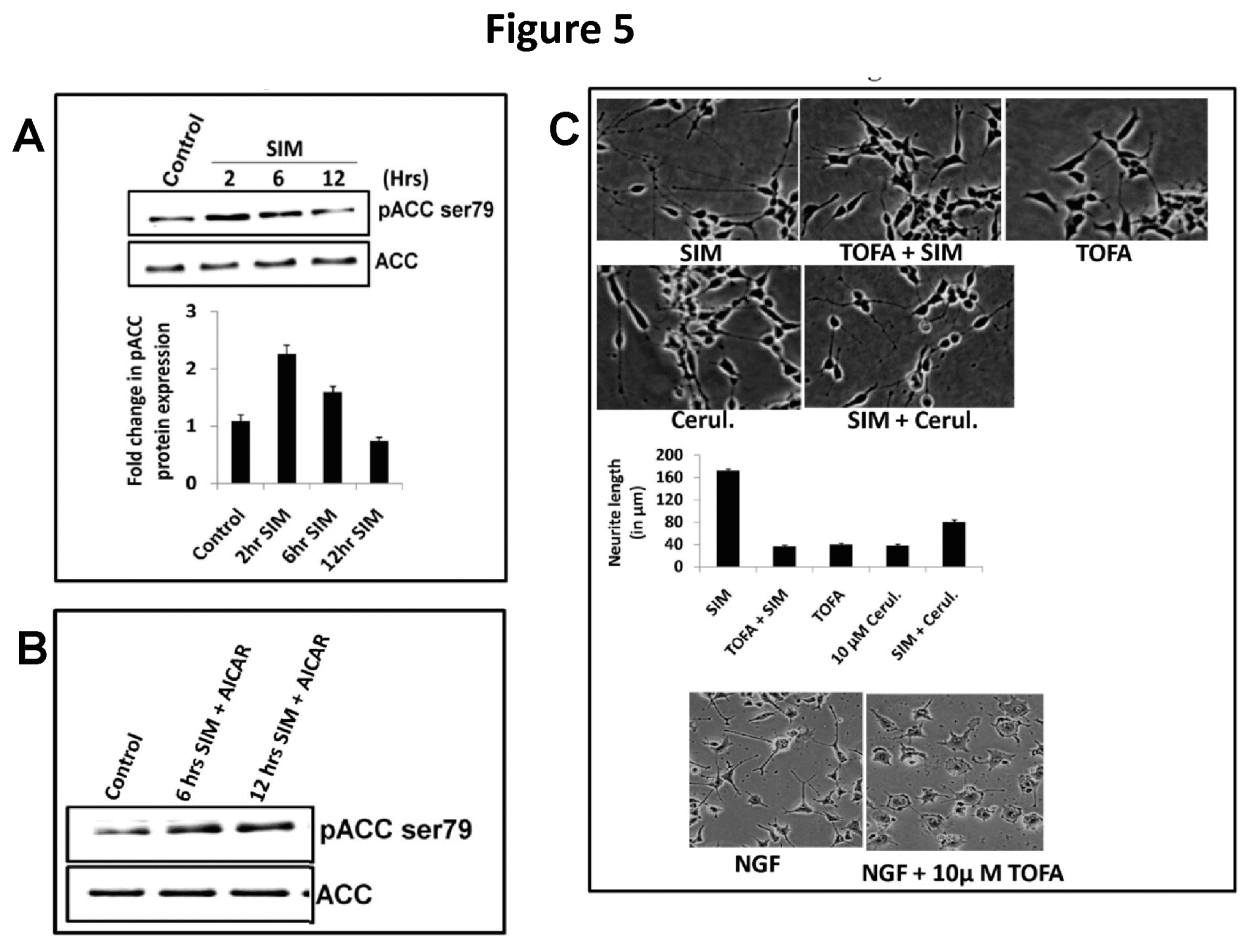
Dephosphorylation of AMPK substrate i.e. acetyl CoA carboxylase (ACC) initiates neuritogenic effect of SIM. **A**) Immunoblot showing SIM induced change in phosphorylation of ACC over a period of 2 hrs, 6 hrs and 12 hrs. SIM led to a significant (*p* < 0.05^*^) increase in pACC (Ser-79) levels at 2 hrs which was followed by a gradual decrease. Total ACC levels showed no change during SIM treatment. Error bar graph shows fold change (normalized to control) in pACC (Ser-79) levels at different time points (mean ± SEM; n = 4). **B**) Immunoblot showing modulation of ACC phosphorylation by SIM follows AMPK pathway. As evident, addition of AICAR (an AMPK activator) increased the phosphorylation of ACC either alone or in presence of SIM till 12 hrs. **C**) Morphology of SH-SY5Y cells under light microscope showing significant (*p* < 0.001) inhibition of SIM induced neuritogenesis in presence of acetyl CoA carboxylase (ACC) inhibitor i.e. 10 µM TOFA and fatty acid synthase (FAS) inhibitor i.e. 10 µM Cerulenin (Cerul.). Error bar graph shows the comparative difference in neurite lengths SIM, TOFA, SIM + TOFA, Cerulenin and SIM + Cerulenin treated cells for a period of 12 hrs (mean ± SEM; N = 50 cells per condition from 3 separate cultures). Morphology of PC12 cells under light microscope in presence of NGF or NGF + TOFA for a period of 3 days. Scale Bar = 100 µm.

Most studies show that supplementing neuroblastoma cells with various classes of fatty acids potentiates the process of neuritogenesis [26,27]. Also, a study showed that over expression of fatty-acyl-CoA synthase (FAS) produces synergistic effect on neuritogenesis with exogenous fatty acids which indicates significant role of endogenous lipid synthesis pathway [39]. FAS is activated by malonyl CoA, a product of acetyl CoA carboxylase (ACC) [40]. ACC is a downstream target of AMPK and the phosphorylation at ser79 by AMPK makes this enzyme inactive. SIM addition led to a significant increase in p-ACC (Ser-79) levels till 6 hrs and thereafter the level of p-ACC (Ser-79) declined drastically even below the control value (Figure **5A**). No change in total ACC levels was seen (Figure **5A**). To further establish the fact that SIM induced dephosphorylation of ACC involves AMPK, we added an AMP analogue i.e. AICAR (5-amino-1-β-D-ribofuranosyl-imidazole-4-carboxamide) to activate AMPK. Addition of AICAR led to a robust increase in p-ACC (Ser-79) levels either alone or in presence of SIM (Figure **5B**). Simultaneously, addition of ACC inhibitor i.e. TOFA led to a drastic decline in SIN (Figure **5C**). We further investigated the effect of ACC inhibitor on NGF treated PC12 cells, a well known model for effect of growth factor on neuritogenesis. As shown in Figure **5C**, TOFA likewise reduced the neurite outgrowth in NGF treated PC12 cells. To establish a link between ACC and fatty acid synthesis, we thus added a Fatty acid synthase (FAS) inhibitor i.e. Cerulenin and observed that SIN was again reduced (Figure **5C**). Thus, our result shows that ACC plays a critical role in determining the extent of neurite extension by SIM in SH-SY5Y cells partially through FAS.

### SREBP-1 pathway is not required but is critical for maintenance of SIM induced neuritogenesis

**Figure 6:**
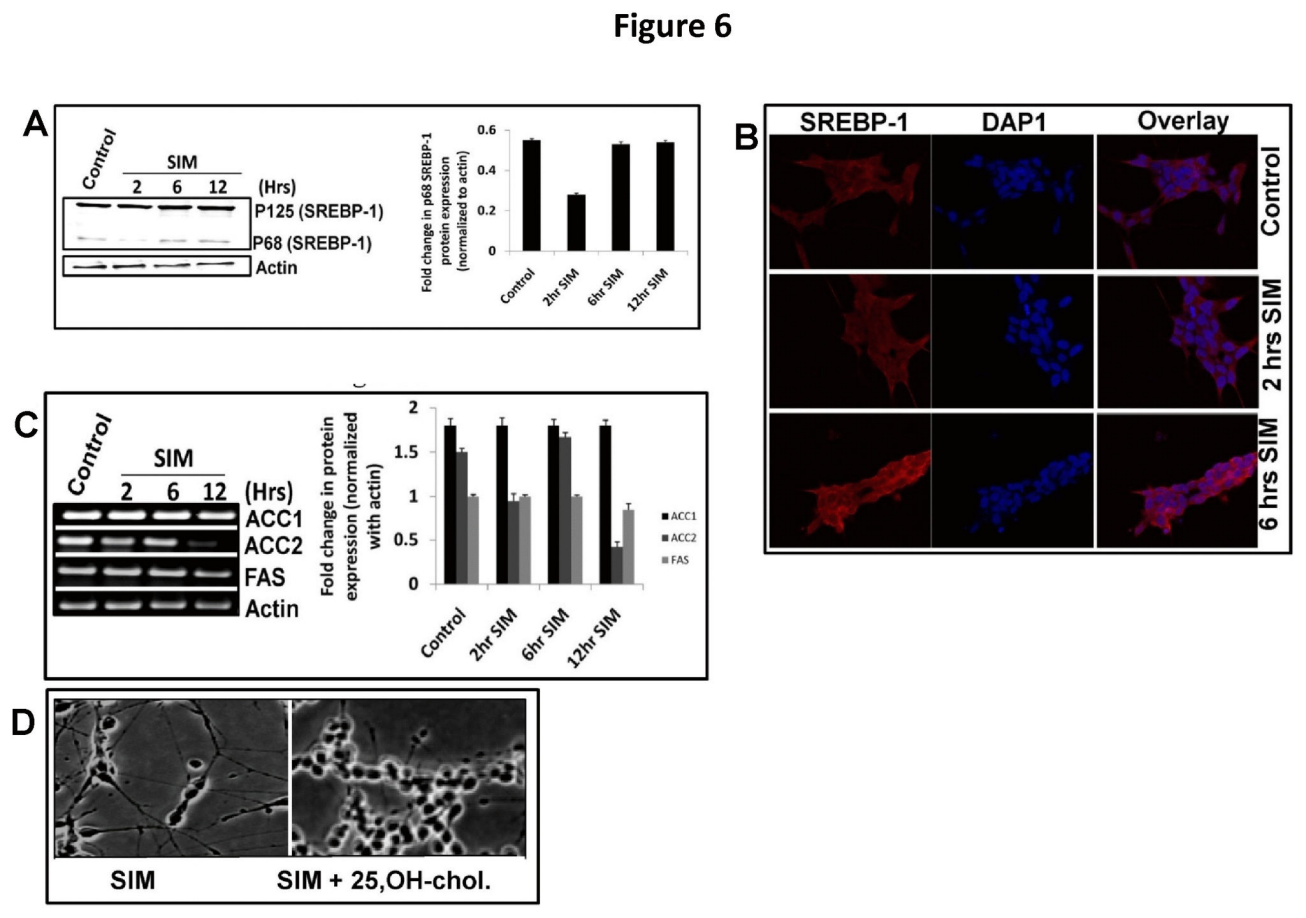
SREBP-1 is dispensable but not required for neuritogenic effect of SIM. A) Immunoblot showing time dependent effect of SIM on SREBP-1 cleavage. p125 represents the immature form whereas p68 represents the mature cleaved form of SREBP-1. SIM led no change on SREBP-1 cleavage except that a significant (*p* < 0.05^*^) decrease was observed at 2 hrs. Error bar graph represents the fold change (normalized to actin) in p68 protein form at different time points in presence of SIM (mean ± SEM; n = 3). **B**) Confocal image at 60 X magnification showing immunofluorescence of SREBP-1 in cytoplasm and nucleus till 6 hrs. An increase in SREBP-1 fluorescence (red) intensity is seen in cytoplasm whereas nucleus represents no change in colour. Nuclei have been stained with DAPI (blue). Error bar graph shows fold change in SREBP (p125) fluorescence before and after SIM treatment. Scale Bar = 10µm. **C**) Semi quantitative RT-PCR showing expression of ACC1, ACC2 and FAS at different time points in presence of SIM. No significant change was observed in transcripts of ACC1. FAS transcripts showed a small decrease at 12 hrs whereas ACC2 transcript showed a significant (*p* < 0.05^*^) decrease at 2 hrs and 12 hrs. Error bar graph represents the comparative fold change (normalized to actin) in ACC1, ACC2 and FAS mRNA levels till 12 hrs in presence of SIM (mean ± SEM; n = 4). **D**) Morphology of SH-S5Y cells under light microscope showing abrogation of SIM induced neuritogenesis in presence of 25-hydroxy-cholesterol during 12 hrs time period. Scale Bar = 100 µm.

AMPK also regulates lipid metabolism through a class of transcription factors known as sterol regulatory element binding proteins (SREBPs) [21,22]. The mature form of SREBPs translocate to nucleus for transcription of its target genes. Among SREBPs, SREBP-1 isoform has been implicated in transcription of ACC1, ACC2 and Fatty Acid Synthase (FAS). We observed that SIM produced no effect on SREBP-1 processing except that a sudden decline was observed in its mature form at 2 hrs (Figure **6A**). On the other hand immature form of SREBP-1 showed slight increase at 6 hrs and 12 hrs which became more visible in confocal imaging (Figure **6A, B**). Simultaneously, RT-PCR analysis showed that SIM did not change transcript level ACC1 whereas FAS levels were decreased slightly at 12 hrs (Figure **6C**). On the other hand ACC2 transcript was significantly reduced at 2 hrs and 12 hrs (Figure **6C**). Consistent with these findings addition of 25-OH-cholesterol (known to inhibit SREBP-1 related functions) led to a complete loss of SIN (Figure **6D**) which implies that constitutive level of SREBP-1 is required for neuritogenic effect of SIM.

## Discussion

In the current study we reveal for the first time interplay between CSC and acetyl CoA carboxylase (ACC; most common substrate of AMPK involved in fatty acid biosynthesis) activity in regulating neuritogenesis and the mechanism involved therein in SH-SY5Y cells

As per published reports on other cells, SIM promoted neuritogenesis in SH-SY5Y cells also [2–4]. Morphologically, neurites showed axon like polarization. Biochemically increased expression of axon specific markers like NFL and β-III provided an evidence for axonal growth. No changes in general neuronal differentiation markers like nestin and GAP43 justify specific effect of SIM on neuritogenesis. Since SH-SY5Y cell is a dopaminergic cells line phosphorylation of Tyrosine Hydroxylase (TH) was also evaluated. SIM addition led to a drastic decline in TH phosphorylation. An earlier report also showed that SIM decreases TH activity in bovine adrenal medullary cells during cardiac neural remodeling [41]. Thus our study also pinpoints the concern about safety of statins like SIM for treating disorders like Segawa’s dystonia, Parkinson’s disease and Schizophrenia.

Reports show that statins redistribute cell surface cholesterol (CSC) in the outer and inner membrane leaflets of plasma membrane in non-neural cells which implies that statins do preserve CSC levels on plasma membrane [5]. As our aim was to just probe this CSC, we used Filipin III reagent which is regarded as a convenient tool for the histochemical identification of unesterfied cholesterol and has greater affinity for cholesterol [42]. Some limitations like rapid photobleaching of the probe, need for free hydroxyl group in a sterol for binding and its binding to other sterols; Filipin III is still used widely as a cholesterol probe [43]. In our study we found that that SIM retained the levels of CSC in SH-SY5Y cells except at 12 hrs where the cholesterol was partly detected in neurites also. Simultaneously, SIM decreased total cellular cholesterol levels. To establish the role of CSC in SIN, we used agents known to perturb surface and intracellular cholesterol pool and found that the retention of CSC is necessary for neuritogenesis. Indeed, applications of agents which perturb cholesterol levels differentially within cells reiterate that CSC promotes neuritogenesis. The neuritogenic effect of SIM was reversed by exogenous cholesterol and mevalonate. Mevalonate is also a precursor for isoprenylation which plays key role in neurogenesis, synapse formation and neuronal differentiation [44]. Contrastingly, studies also demonstrate that increased levels of isoprenoids may contribute to neuronal dysfunction observed in aging and certain neurodegenerative diseases [45,46]. In our study inhibition of geranylgeranyl transferase (GGT) inhibited SIN which reinforces the fact that isoprenylation might be a contributing or alternate factor to govern SIN. However, few studies show that inhibition of geranylgeranyl transferase (GGT) mimics the effects of statin in other neuroblastoma cells and therefore warrants further investigation [3,47]. Furthermore, we also found that SIN was not as a result of sphingolipid perturbation. Importantly, addition of exogenous sphingolipid like sphingomyelin produced negative effect on SIN.

In plasma membrane, CSC exists in discrete domains known as lipid rafts which play an important role in neuronal differentiation, synaptogenesis and axonal guidance [48]. We found that SIM maintained the lipid raft integrity as indicated by marked increase and localization of Flotillin-2 therein. This was accompanied by enhanced phosphorylation of various p-RTKs which represent functional markers of cholesterol rich lipid rafts. Based on the results reported here it is apparent that the effect of mevastatin reported earlier leading to an induction of EGFR phosphorylation might be related to the stabilization of rafts [49]. RTKs trigger a wide range of downstream signaling cascades predominantly through ERK, p38, JNK and PI3K signaling. However, we found that SIM activated mostly PI3K/Akt survival pathway and enhanced phosphorylation of HSP27 protein involved in stress. Similar effect of SIM on Akt and HSP27 was reported as neuroprotective in axotomized retinal ganglion cells *in-vivo* [50]. Importantly, inhibition of PI3K activity with both reversible and an irreversible inhibitor indicated that SIN is dependent on the modulation of PI3K/Akt pathway.

PI3K/Akt pathway exerts pleotropic effect on cells, including control of lipid metabolism by inhibiting phosphorylation of a metabolic sensor known as AMP-activated protein kinase (AMPK) [32]. AMPK is widely expressed in brain and is required to meet metabolic and energy demands in neurons [12]. Importantly, neuritogenesis is also considered as an energy demanding process. We found that SIM had dual effect on AMPK phosphorylation i.e. a transient increase followed by rapid reversal to basal level. However, addition of AMPK activator revealed that AMPK is negative regulator of neuritogenesis. Recently, a study also demonstrated that AMPK acts as a negative regulator of axonal growth [14]. However, the study did not provide any mechanism which links AMPK to neuritogenesis. Furthermore, taking into account the importance of AMPK in cell survival and function; we believe that SIM induced transient increase in AMPK phosphorylation might be a response for some biological event required for cellular homeostasis. Surprisingly, we noticed that SIM induced dephosphorylation of AMPK was independent of PI3K activity. Several lines of evidence suggest that simultaneous phosphorylation at serine and threonine residues of Akt are necessary for its full activation [33]. Our p-MAPK array data did not reveal this difference; however immunoblot for ser-Akt and Thr-Akt showed that SIM enhanced only threonine phosphorylation of Akt.

To elucidate the mechanism behind AMPK dephosphorylation we suspected involvement of Protein phosphatase 2A (PP2A), known to regulate phosphorylation of AMPK and MAPKs [34–36]. Tyrosine phosphorylation of PP2A in its catalytic subunit has been shown to decreases its activity [37]. Strikingly, SIM decreased tyrosine phosphorylation of PP2A and inhibition of PP2A activity not only enhanced AMPK phosphorylation but also counteracted the neuritogenic effect of SIM. Though, neuritogenic effect of PP2A has been shown in other studies; however, our study demonstrates for first time the nexus between PP2A and AMPK in regulating neurite outgrowth [51–53].

Neuritogenesis requires continuous supply of phospholipids synthesized from fatty acids [15,16]. Importantly, AMPK controls fatty acid biosynthesis by regulating the phosphorylation of acetyl CoA carboxylase (ACC) [12,13]. ACC produces malonyl-CoA substrate for fatty acyl CoA synthase (FAS), a committed enzyme for fatty acid biosynthesis [20]. Interestingly, a study showed that lovastatin enhances fatty acid biosynthesis in cultured hepatocytes by activating ACC [54]. Our study is thus consistent with the enhancement of ACC activity in SH-SY5Y cells by SIM as indicated by a decrease in its phosphorylation. However, the rate at which ACC got dephosphorylated was not so fast as compared to AMPK. This led us to doubt whether AMPK lies upstream to ACC and indeed addition of an AMP analogue confirmed that SIM modulated phosphorylation of ACC occurs through AMPK. Simultaneously, we found that SIN was antagonized by inhibition of ACC and FAS activity. However, inhibition of ACC activity produced more drastic effect on SIN. We argue that malonyl CoA not only controls fatty acid metabolism but also has an important signaling function through its allosteric inhibition of carnitine palmitoyltransferase 1, the enzyme that normally exerts flux control over mitochondrial β-oxidation [55]. Thus our study puts forward an important link between SIM and mitochondrial β-oxidation pathway which further needs an independent investigation. Remarkably, we also found that ACC activity is important for NGF induced neurite outgrowth in PC12 cells.

Homeostasis of cellular sterol levels plays an important role in determining the maturation of a class of transcriptional factors known as Sterol Responsive Element Binding Protein’s (SREBPs), bound to endoplasmic reticulum as inactive precursor [21]. SREBPs exist in two major isoforms: SREBP-1 and SREBP-2. Out of these, SREBP-1form is involved in transcription of genes like ACC and FAS. Furthermore, it is suggested that AMPK may affect the processing of SREBP-1 by an unknown mechanism [22]. We found that SIM neither changed SREBP-1 processing nor affected transcript levels of its target genes i.e. ACC1 and FAS. On the other hand ACC2 transcripts showed an oscillatory trend. The importance of differential regulation of ACC isoforms during neuritogenesis is evident from the fact that ACC1 is involved in fatty acid biosynthesis whereas ACC2 activates fatty acid catabolism [21,40]. However, inhibition of SREBP-1 activity by 25-hydroxycholesterol revealed that constitutive level of SREBP-1 is essential for maintaining SIN and warrants further investigation.

**Figure 7:**
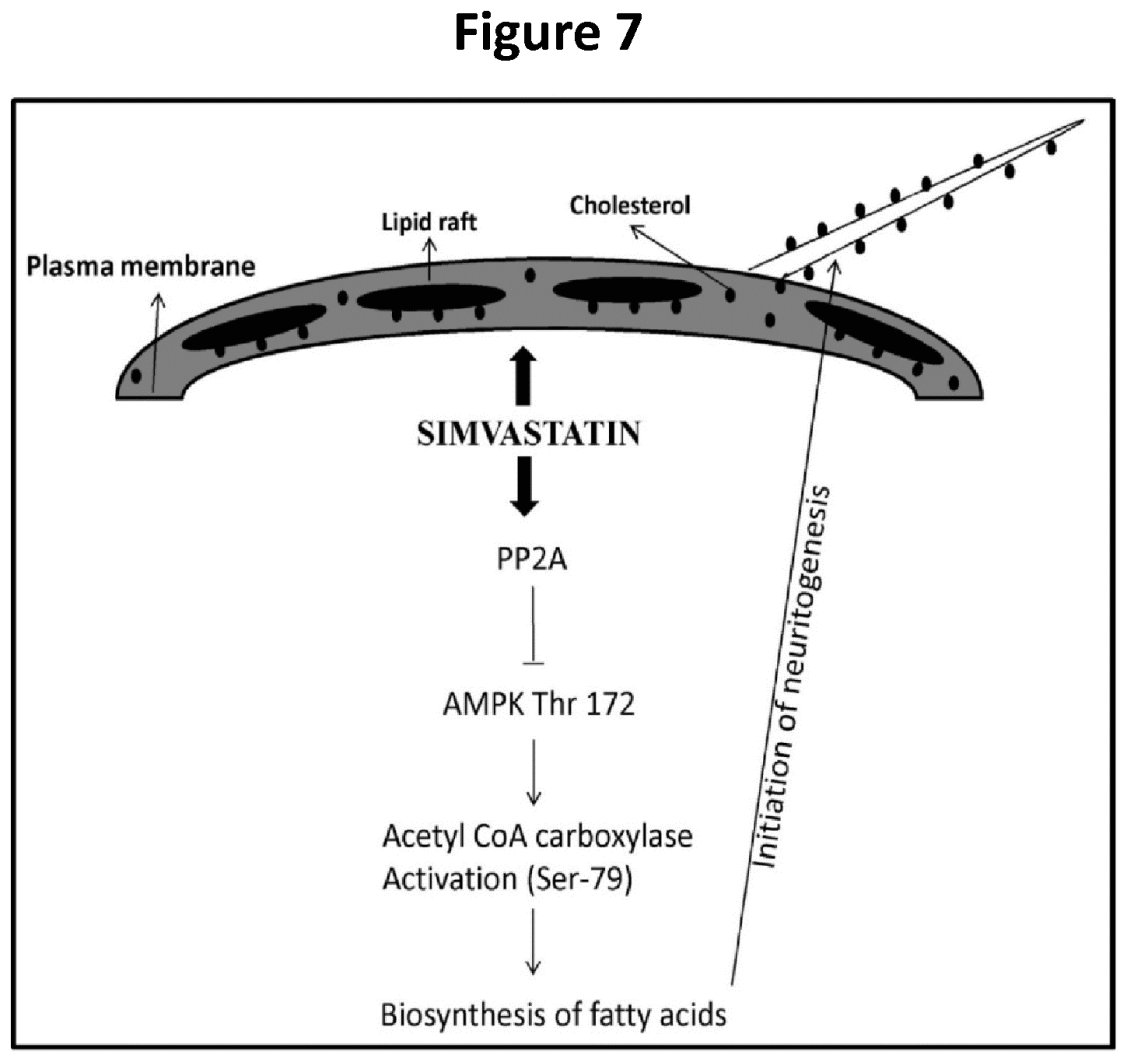
Schematic representation of SIM (simvastatin) induced neuritogenesis pathway. SIM treatment to SH-SY5Y cells promotes retention of cell surface cholesterol (CSC) which regulates cellular function by stabilizing lipid raft signaling and maintenance of neurite outgrowth. Simultaneously, depletion of cholesterol by SIM activates an intracellular phosphatase i.e. Protein phosphatase 2A (PP2A) which inactivates AMPK by dephosphorylating at Thr 172. This is followed by activation of acetyl CoA carboxylase (ACC) through dephosphorylation at Ser-79. New fatty acids get synthesized which act as precursors for neurites during elongation process.

In conclusion, this study unravels a co-ordinated action of CSC and ACC in SIN (Figure **7**). We highlight two major events involved during SIN: 1) retention of CSC which acts as stabilizer to orchestrate signaling events necessary to promote neuritogenesis; 2) initiation of fatty acid biosynthesis by ACC activation through PP2A phosphatase dependent de-phosphorylation of AMPK.
